# Uranium from German Nuclear Power Projects of the 1940s— A Nuclear Forensic Investigation

**DOI:** 10.1002/anie.201504874

**Published:** 2015-09-07

**Authors:** Klaus Mayer, Maria Wallenius, Klaus Lützenkirchen, Joan Horta, Adrian Nicholl, Gert Rasmussen, Pieter van Belle, Zsolt Varga, Razvan Buda, Nicole Erdmann, Jens-Volker Kratz, Norbert Trautmann, L Keith Fifield, Stephen G Tims, Michaela B Fröhlich, Peter Steier

**Affiliations:** European Commission, Joint Research Centre (JRC) Institute for Transuranium Elements (ITU), Postfach 2340, 76125 Karlsruhe (Germany); Institut für Kernchemie, Universität Mainz Fritz-Strassmann Weg 2, 55128 Mainz (Germany); Department of Nuclear Physics, Research School of Physics and Engineering The Australian National University, Canberra ACT 2601 (Australia); Universität Wien, Fakultät für Chemie Institut für Anorganische Chemie, Althanstrasse 14, 1090 Vienna (Austria); Universität Wien, Fakultät für Physik Isotopenforschung und Kernphysik, Währinger Strasse 17, 1090 Vienna (Austria)

**Keywords:** Heisenberg, Werner, mass spectrometry, nuclear forensics, uranium, Wirtz, Karl

## Abstract

Here we present a nuclear forensic study of uranium from German nuclear projects which used different geometries of metallic uranium fuel.3b,d, 4 Through measurement of the ^230^Th/^234^U ratio, we could determine that the material had been produced in the period from 1940 to 1943. To determine the geographical origin of the uranium, the rare-earth-element content and the ^87^Sr/^86^Sr ratio were measured. The results provide evidence that the uranium was mined in the Czech Republic. Trace amounts of ^236^U and ^239^Pu were detected at the level of their natural abundance, which indicates that the uranium fuel was not exposed to any major neutron fluence.

Soon after the discovery of nuclear fission^1^ its potential as a useful source of energy was realized. Within a few months, characteristic properties of the fission process were identified^2^ and in 1942, the first manmade self-sustaining chain reaction was achieved. As a result, nuclear research projects were initiated in the United States and Germany.^3^ Whether the German nuclear projects had a military dimension or were rather aimed at the construction of an “atomic” reactor for energy production—or both—has previously been discussed.3b–d, 5 The experiments on neutron multiplication in different fuel geometries were conducted by two groups headed by W. Heisenberg at the Kaiser Wilhelm Institute (KWI) for Physics, Berlin, and by K. Diebner of the Army Ordnance. The Heisenberg group used alternating layers of fuel and moderator, e.g., uranium plates (with K. Wirtz in Berlin), while the Diebner group used cubes.3d, 4a,b After a series of experiments, Heisenberg recognized the superior neutron economy of the cube design and followed this approach.

The last experiment, called B8, took place in March 1945,3a after the relocation of the Kaiser Wilhelm Institute for Physics to Hechingen, near Haigerloch (Southern Germany). Instead of the previous plate geometry, 664 uranium metal cubes from the Diebner group (ca. 1.5 tons of uranium) were used as fuel (Figure 1),4a resulting in a neutron multiplication factor of 6.7. Criticality was expected for a reactor volume about 50 % larger.4a

**Figure 1:**
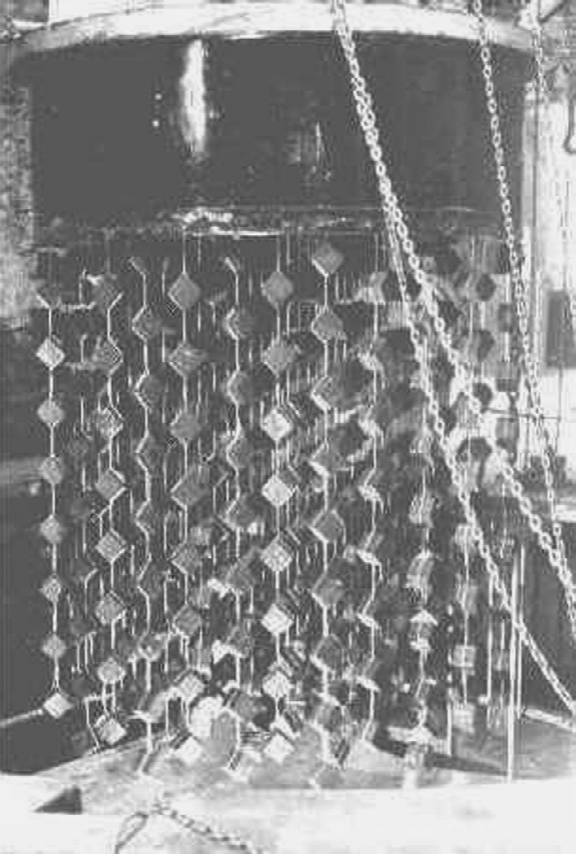
Photograph of the uranium pile (B8) showing 664 cubes.4a

The majority of the uranium cubes were recovered in April 1945 by the ALSOS mission.3a,b, 4c Some twenty years later, several cubes (called “Heisenberg cubes” below) resurfaced in Southern Germany. In 1998, one of them was examined by the German Federal Office for Radiation Protection (BfS)^6^ and was sent to the Institute for Transuranium Elements (ITU) for nuclear forensic investigations to verify its authenticity. Two years later, a uranium metal plate was retrieved at the Max Planck Institute (MPI) for Nuclear Physics in Heidelberg. The material (called the “Wirtz plate”) was attributed to the experiments by the Heisenberg–Wirtz group and was sent to ITU for further investigation.

The experiments presented here are structured as a nuclear forensic investigation^7^ and address the following questions:

What are the macroscopic parameters and the elemental composition of the material? What is its age, in other words, when was the last chemical separation of uranium? Is the uranium enriched in ^235^U? Was the uranium exposed to some major neutron fluence? What is the origin of the uranium ore used for production of the uranium metal?

To answer these questions, various characteristic parameters were determined, including the isotope ratios ^230^Th/^234^U, ^234,235,236^U/^238^U, ^239^Pu/^238^U, and ^87^Sr/^86^Sr, as well as the rare-earth elemental (REE) abundance pattern. Several of these data were also determined for a sample of ammonium diuranate (yellow cake) from the Hahn and Strassmann laboratory at KWI for Chemistry in Berlin (called “Hahn YC”) and for uranium ore and ore concentrate (UOC) samples from Joachimsthal/Jáchimov (Ore Mountains region of Bohemia and Saxony) and the Shinkolobwe mine (the former Belgian Congo) as potential uranium sources. The results provide an experimental contribution to the discussion of German nuclear projects during the early 1940s.

## Experimental Section

**Figure 2:**
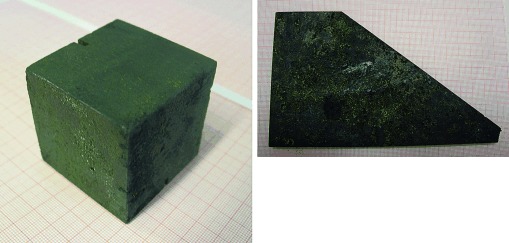
Photographs of the analyzed samples. Left: Heisenberg cube I (5 cm×5 cm×5 cm). Right: Wirtz plate (trapezoid with 18 cm base, 1 cm thickness; broken corner on the lower right).

Three uranium metal samples were investigated: 180 mg powder from an uranium cube received from BfS (Heisenberg cube I), a 1 mm thick, 47.8 g metal piece sliced off an uranium cube from the “Atomkeller-Museum” in Haigerloch (Heisenberg cube II), and several small pieces (weights vary) sawed off from the Wirtz plate (Figure 2). Fewer analytical measurements were performed with the Heisenberg cube I due to the small size of the sample; all results reported here are from cube II, unless stated otherwise.

Uranium samples were dissolved in nitric acid and chemically separated^8^ in order to preconcentrate the elements of interest (Sr, Th, U, and Pu) and analyzed by the following methods: thermal ionization mass spectrometry (TIMS) for ^234,235,236,238^U,^9^ accelerator mass spectrometry (AMS) for ^236^U,^10^ multicollector inductively coupled mass spectrometry (MC-ICP-MS) for strontium isotopes,8a sector-field ICP-MS for rare-earth elements8b and age determination, alpha-spectrometry (AS) for age determination,^11^ and resonance ionization mass spectrometry (RIMS) for ^239^Pu.^12^ All uncertainties quoted are expanded uncertainties with a coverage factor of *k*=2, unless stated otherwise.

## Macroscopic Investigation

The Heisenberg cube has a side length of 5 cm and a mass of 2.4 kg. The cube has two notches at the middle of two opposite edges (Figure 2, left) for the fixing wires (cf. Figure 1). The Wirtz plate is of trapezoidal geometry with a 18 cm base, 1 cm thickness, and a missing corner tip of about 1 cm (Figure 2, right).

The Heisenberg cubes correspond to the description of the uranium metal cubes produced by Degussa in 1943–1944 and used in the G3 and B8 experiments.4a The Wirtz plate is likely a fragment of a larger metal plate that had been produced for and used in earlier experiments (e.g. B6, B7).4c The geometries therefore indicate that both samples analyzed were produced for the reactor experiments performed by the Heisenberg and Diebner groups.

## Uranium Isotopic Analysis

Uranium isotope ratios were measured for the Heisenberg cube, the Wirtz plate, and the Hahn YC sample. The ^235^U/^238^U abundance ratios in the three samples agree well (Table 1) and correspond to the natural value,^13^ that is, samples were not enriched in ^235^U. This is in line with the German enrichment technology level at that time^5^ which did not reach beyond the experimental stage.^14^ The ^234^U abundance may show small variations due to chemical fractionation effects in nature, e.g., preferential leaching of ^234^U after alpha-recoil. Hence, it serves as useful parameter in geolocation. No significant difference in the ^234^U/^238^U ratio was observed. This indicates that the uranium source materials (i.e. the ore) originate very likely from the same mine for all three investigated samples.

**Table 1:** ^235^U/^238^U and ^234^U/^238^U isotope abundance ratios determined by TIMS.

Sample	^235^U/^238^U	^234^U/^238^U
Heisenberg cube	(7.2526±0.0053)×10^−3^	(5.4809±0.0067)×10^−5^
Wirtz plate	(7.2531±0.0053)×10^−3^	(5.4781±0.0074)×10^−5^
Hahn YC	(7.2584±0.0103)×10^−3^	(5.4819±0.0120)×10^−5^

## Isotopic and Elemental Analysis of Minor Constituents

The isotopic composition of minor constituents (e.g. Sr) in uranium ores provides clues as to the geolocation of the processed natural uranium. Typically, a fraction of the minor constituents passes through mineral processing into the product material with its original isotopic composition preserved. The same holds for an elemental pattern, such as the REE, if the chemical behavior of the elements is similar to that of uranium.

The ^87^Sr/^86^Sr ratio varies by almost 10 % in UOCs, typically from 0.70 to 0.76,8a and depends on the type and age of the uranium ore and its Rb/Sr ratio. Also, the REE pattern may serve to distinguish between different mines and deposit types.8b Such signatures include the shape of the distribution pattern and an europium or cerium abundance anomaly.

The ^87^Sr/^86^Sr ratios obtained for the Heisenberg cube, the Wirtz plate, and the Hahn YC samples are 0.7037(33), 0.7078(10), and 0.7071(30), respectively, and agree within experimental uncertainty. The ^87^Sr/^86^Sr value for the Joachimsthal ore is in the range between 0.703 and 0.707,^15^ whereas UOC from the former Belgian Congo (archive sample at ITU) has a higher value of 0.71101(8). The Sr isotope abundance ratios of the uranium samples are consistent with the Joachimsthal ore values.

**Figure 3:**
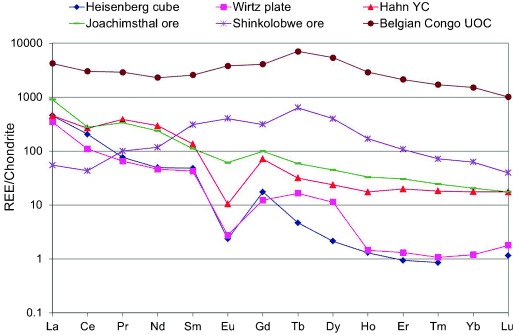
The rare-earth element patterns of the investigated uranium samples.

The REE abundances, normalized to chondrite values, are shown in Figure 3 for the uranium metal samples and the Hahn YC. Two ore samples from Joachimsthal and Shinkolobwe, as well as UOC from the former Belgian Congo were measured for comparison, since at that time, Germany had access to uranium minerals from both regions.3a These two uranium deposits are of different geological formations (Joachimsthal: granite-related vein deposit, Shinkolobwe: unconformity related/metamorphic deposit^16^). The uranium metal samples, the Joachimsthal ore, and the Hahn YC have similar REE patterns (pronounced Eu anomaly and lower concentration towards the heavier REE), whereas the patterns of Shinkolobwe ore and UOC from the former Belgian Congo are distinctly different (bell-shaped curve, no Eu anomaly). This is strong evidence that the uranium ore used for production of the Heisenberg cube, the Wirtz plate, and the Hahn YC was mined in the Joachimsthal region.

## ^236^U and ^239^Pu as Indicators of Neutron Fluence

^236^U and ^239^Pu are produced in reactor systems through neutron capture by ^235^U and ^238^U. As the ^236^U abundances of the Heisenberg cube and the Wirtz plate were below the TIMS detection limit, small samples were measured by AMS^10^, ^17^ at ANU, Canberra, and at UW, Vienna. The ^236^U/^238^U ratios (Table 2) are on the order of 10^−10^ for the cube, the plate, and the Hahn YC. The ratios are in the range typical for uranium ores10b between 10^−12^ and 3×10^−10^. The ratios in the uranium metals and the Hahn YC indicate that ^236^U is of natural origin. The natural ^236^U/U ratio is determined by the thermal neutron flux, which can be expected to be inhomogeneous even in the same uranium deposit. Literature values for Joachimsthal ore (Jachymov, Czech Republic) range from (3.18±0.43)×10^−11^ 10b to (9.0±2.0)×10^−11^.^18^ The values obtained for all three samples are in agreement with this range; however, the slight difference in the isotopic ratios observed for Heisenberg cube and Wirtz plate may suggest that the materials did not come from the same processing batch.

**Table 2:** ^236^U/^238^U isotope abundance ratios and ^239^Pu/U concentrations (g/g U) for various samples as determined by AMS and by RIMS. The AMS and RIMS data are average values of 2 to 6 independent measurements. All uncertainty values with *k*=1.

Sample	^236^U/^238^U [×10^−10^]	g ^239^Pu/g U [×10^−14^]
Heisenberg cube	0.91±0.05^[a]^ 1.00±0.04^[b]^	1.6±0.8^[c]^
Wirtz plate	1.10±0.05^[a]^ 1.11±0.03^[b]^	1.4±0.7^[c]^
Hahn YC	1.02±0.03^[b]^	–
Joachimsthal ore	–	8.5±2.8^[c]^

[a] Data from ANU, Canberra.

[b] Data from UW, Vienna. [c] Data from UM, Mainz.

The ^239^Pu abundances were measured by RIMS because this method has higher sensitivity than TIMS. The ^239^Pu/U ratios (Table 2) of the cube and the plate are in excellent agreement in the range of (1–2)×10^−14^.

The Joachimsthal uranium ore sample has a ^239^Pu/U ratio of ca. 10^−13^, which is six time higher than that of the metal samples and of the same order as the ^239^Pu/U ratios in natural uranium ores.10b For metal production, the uranium material was purified from decay products of uranium including thorium. At that time, no information about plutonium and its chemical behavior was available in Germany. It can be assumed that in the purification process, a large fraction of the plutonium was removed together with thorium, provided it was in the tetravalent state. ^239^Pu built up by neutron capture during the B8 reactor experiments is negligible, likely even for the case of a hypothetical criticality: Using the initial neutron flux from a RaBe source4a together with a contribution from ^238^U spontaneous fission neutrons, the reported B8 neutron multiplication of 6.7,4a and an assumed one-week irradiation time, one obtains a ^239^Pu/U fraction of about 10^−16^. Assuming a higher neutron multiplication up to criticality—for example, under conditions reported for Fermi’s CP-1 reactor in Chicago with 4.5 min operation of 0.5 W4a—one obtains a ^239^Pu/U ratio of 7×10^−16^. The majority of the ^239^Pu found in the Heisenberg cube and the Wirtz plate is, therefore, most likely of natural origin; there is no evidence that plutonium in the Heisenberg cube was formed to a larger extent by neutron irradiation.

## Age Determination

The age of uranium materials, determined from the measured ^230^Th/^234^U ratio, reflects the time when the last chemical treatment of uranium (separation of impurities and decay products) was performed. For metal samples this will be the date of casting. In that sense, the Heisenberg cubes were produced in the second half of 1943, while the Wirtz plate was produced some three years earlier (Table 3). The date of the metal cube production is consistent with literature information on the change of reaction design, moving from an alternating layer approach to uranium cubes suspended in heavy water.4c In conclusion, the age determination confirms the authenticity of the two uranium metals and provides experimental evidence of the production dates.

**Table 3:** Age determination of uranium materials by isotope dilution alpha spectroscopy (ID-AS) and mass spectrometry (ICP-MS). Production date with 1 *σ* uncertainty, *k*=1.

Sample	ID-AS	ICP-MS
Heisenberg cube I	Dec 1943±1.5 a	Sept 1943±2.0 a
Heisenberg cube II	June 1944±0.8 a	Sept 1943±0.5 a
Wirtz plate	Aug 1940±0.3 a	–

## Conclusion

Samples of uranium metals were analyzed and their authenticity as “Heisenberg cubes” and a “Wirtz plate” from German nuclear power projects of the early 1940s was confirmed. The samples are among the oldest manmade uranium items produced for the purpose of studying neutron multiplication up to a self-sustained chain reaction. The authenticity was confirmed by 1) comparison of macroscopic sample properties with literature information and 2) determining the production date (called “age”) of the uranium as 1940 for the plate and 1943/44 for the cubes. The uranium was mined in the Joachimsthal region rather than in the former Belgian Congo, as shown by the abundance pattern of rare-earth elements. The isotopes ^236^U and ^239^Pu were used as neutron fluence monitors. The measured abundances are consistent with natural values and do not indicate a major contribution due to a neutron fluence during reactor experiments.

## Supporting Information

As a service to our authors and readers, this journal provides supporting information supplied by the authors. Such materials are peer reviewed and may be re-organized for online delivery, but are not copy-edited or typeset. Technical support issues arising from supporting information (other than missing files) should be addressed to the authors.

miscellaneous_information
