# Supplementary material for: Uranium from German Nuclear Power Projects of the 1940s— A Nuclear Forensic Investigation
Source: Angew Chem Int Ed Engl. 2015 Sep 7;54(45):13452–6. doi: 10.1002/anie.201504874 (PMC4678420; doi:10.1002/anie.201504874)
Supplement: Supplementary file 1 [file anie0054-13452-sd1.pdf]

Angewandte Chemie
Eine Zeitschrift der Gesellschaft Deutscher Chemiker

Supporting Information

Uranium from German Nuclear Power Projects of the 1940s—A Nuclear Forensic Investigation

Klaus Mayer, Maria Wallenius, Klaus Lützenkirchen, Joan Horta, Adrian Nicholl, Gert Rasmussen, Pieter van Belle, Zsolt Varga, Razvan Buda, Nicole Erdmann, Jens-Volker Kratz, Norbert Trautmann, L. Keith Fifield, Stephen G. Tims, Michaela B. Fröhlich, and Peter Steier*

anie_201504874_sm_miscellaneous_information.pdf

Supporting information

Methods

The analytical scheme for the uranium samples analyzed here is shown in Scheme 1. The scheme is similar to nuclear forensic investigations of seized samples, however, it was adapted to the case specific requirements. Some techniques, such as AMS and RIMS, have not been employed before for nuclear forensic investigations.

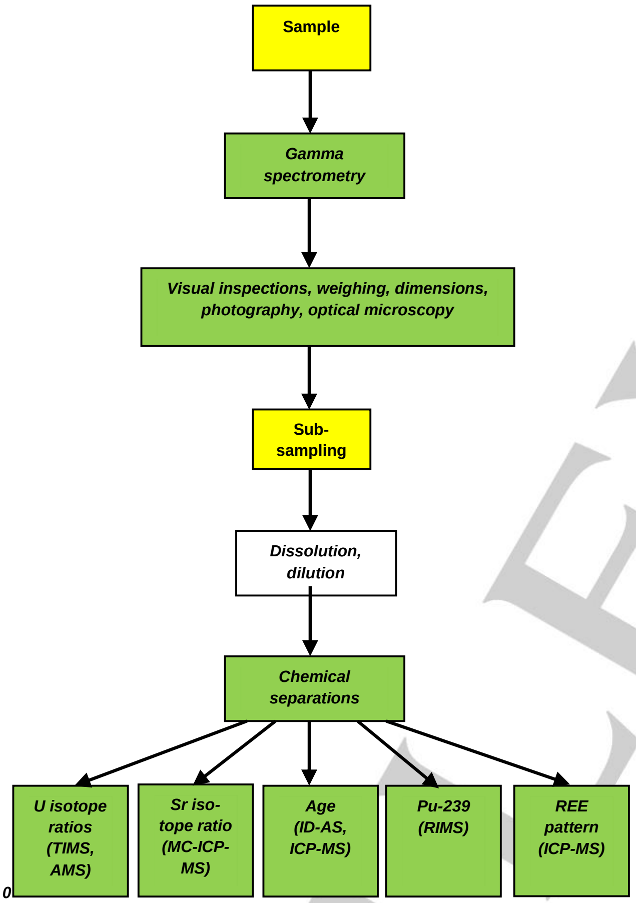

Scheme 1. Scheme for the analysis of uranium samples.

Uranium isotope measurements for mass numbers 234, 235, 236 and 238 were carried out by thermal ionization mass spectrometry (TIMS) using a Triton instrument (Thermo Corporation). A double filament assembly was used and the instrument was run in a modified total evaporation mode [9]. Thus, the ion currents of all isotopes were recorded simultaneously and the signals were integrated over the entire measurement time. This eliminates time dependent isotope fractionation effects. The ^{234}U and ^{236}U ion currents were recorded using micro channeltrons (continuous dynode electron multiplier), while Faraday Cups were used for measuring the signals arising from ^{235}U and ^{238}U. The ^{236}U ion current was below the detection limit of $5 \cdot 10^{-9}$ for the ^{236}U/^{238}U ratio. Therefore, further investigations were carried out using accelerator mass spectrometry (AMS) [10, 17].

In order to determine the REE abundance pattern, a group separation of the REE from the uranium matrix was applied [8b]. The REE were separated using extraction chromatography by the selective retention of trivalent lanthanides on a TRU^{TM} resin in 3 M nitric acid medium. After washing the column and removal of the non-retaining matrix components, the REE were stripped from the column using HCl. After the addition of 200 µl ultra-pure HNO_3 to the final fractions, the samples were evaporated to almost complete dryness on a hot-plate in order to destroy the organic resin residual. The residue was dissolved in 1 ml of 2% (m/m) ultra-pure nitric acid while heating slightly. After the weighing of the final fractions and the addition of a rhodium internal standard, the samples were analyzed by ICP-MS using external calibration.

The age of the uranium samples, i.e., the time elapsed since the last separation of uranium from its daughter nuclides, was determined [11] from the daughter/parent isotope ratio ^{230}Th/^{234}U. Aliquots of the respective uranium solutions were spiked with ^{233}U for the ^{234}U analysis, and with ^{228}Th and ^{232}Th for the ^{230}Th analysis by alpha spectrometry and ICP-MS, respectively. Uranium and thorium were separated using TEVA ion chromatography resin (Eichrom Technologies Inc., Darien, Illinois, USA). Thorium fractions were measured by α-spectrometry and ICP-MS, uranium isotopes by isotope dilution mass spectrometry (IDMS) using a TIMS mass spectrometer (MAT261, Thermo Finnigan, Bremen, Germany). For comparison, the ^{230}Th/^{234}U ratio was measured also directly by ICP-MS without any chemical separation and spiking.

For the separation (and pre-concentration) of strontium from the uranium samples, a sequential extraction chromatographic

separation method was applied, which enables the simultaneous separation of Pb and Sr from one aliquot [8a]. In short, ultrapure water was added to the dissolved stock solutions of the uranium in order to adjust the nitric acid concentration to approximately 2-3 M. The extraction chromatography resin (Sr Resin™ 100-150 µm, Eichrom Technologies Inc., Darien, Illinois, USA) was cleaned with 8 M HCl before the sample was loaded on the column. After sample loading, the matrix components were stripped from the resin using 2 M HNO_3, 7 M HNO_3, and again 2 M HNO_3. Thereafter, strontium was eluted from the column using 0.05 M HNO_3. Ultra-pure HNO_3 was added to the strontium fraction which was then evaporated to almost complete dryness on a hot-plate in order to destroy any residual organic resin. The evaporated fractions were dissolved in 2% (m/m) ultra-pure nitric acid while heating slightly. The final sample fraction was analyzed by multi-collector ICP-MS equipped with 11 Faraday collectors and 3 discrete dynode electrode multipliers (NuPlasma, Nu Instruments, Oxford, UK).

The ^{239}Pu content of the uranium samples was determined by resonance ionization mass spectrometry (RIMS). For this, plutonium is selectively ionized via a three step resonant photo-excitation with subsequent mass separation yielding detection limits in the range of 10^6 atoms in the sample with complete suppression of isobaric interferences [12]. Prior to the RIMS measurements, plutonium was separated from the metallic uranium and the uranium ore samples as follows: The uranium metal samples were first etched with hot 8 M HNO_3 in order to remove any potential surface contamination. After this, the samples were dissolved in hot 8 M HNO_3 and the solutions were divided into three aliquots. Each of the aliquots was spiked with a known amount of ^{244}Pu for the determination of the overall yield. From the solutions $(NH_4)_2U_2O_7$ was precipitated by adding conc. NH_3. The precipitate was re-dissolved after filtration in 4 M HNO_3. This solution was passed through a chromatographic column (TEVA-Resin SPS, preconditioned with 4 M HNO_3). After a washing step with 4 M HNO_3, plutonium was eluted with 0.5 M HCl. The eluate was evaporated to dryness, dissolved in 2M HNO_3 and a small amount of $NaNO_2$ was added in order to obtain Pu(IV). Then, Pu was sorbed on an anion exchange chromatographic column (Bio-Rad AG MP-1) and after a washing step with 2 M HNO_3 it was eluted with 0.36 M HCl/0.025 M HF. The eluate was evaporated to near dryness, H_2SO_4 was added and again evaporated. The residue was dissolved in 20 % $(NH_4)_2SO_4$ at pH 1.5 and transferred in an electrolysis cell where plutonium was deposited on a tantalum backing. The resulting filaments were covered with a thin layer of metallic titanium for the reduction of plutonium to the elementary state and subsequently measured by RIMS.

AMS uses a particle accelerator as a mass spectrometer to determine $^{236}U/^{238}U$. At both ANU and UW (VERA accelerator), UO^- ions from the solid sample are produced in the cesium sputter source, analyzed for mass, and accelerated to MeV energies in a tandem accelerator, where they are stripped to multiply positive charge states. The ANU tandem was operated at ~4 MV terminal voltage, while VERA uses 3 MV. Molecules are broken up in this process, and a second mass analysis can separate $^{236}U^{5+}$ without molecular interference. Time-of-flight setups are used at both laboratories to further suppress background.

For the ^{236}U determination at VERA, the Heisenberg cube and Wirtz plate samples were slightly leached with 3M HNO_3 to remove any potential anthropogenic contamination from the surface and then rinsed twice with Milli-Q (18 MΩ·cm) water. The Hahn yellow cake sample was divided into two sub-samples, of which one was chemically separated by UTEVA® resin (Eichrom Technologies, Inc.) following the procedure given in Ref. [17]. The samples were transferred to small quartz tubes and combusted at 800°C for 2 h in a muffle furnace and pressed in suitable holders for AMS measurement. While only one sample of the separated yellow cake was prepared, three independent aliquots were prepared for the Heisenberg cube, the Wirtz plate, and the untreated Hahn yellow cake. The aliquots were measured in four independent AMS measurement runs. All aliquots showed good agreement within uncertainties, thus the quoted results are the average of the values obtained on each sample.

WILEY-VCH
